# Additive interactions of unrelated viruses in mixed infections of cowpea (*Vigna unguiculata* L. Walp)

**DOI:** 10.3389/fpls.2015.00812

**Published:** 2015-10-01

**Authors:** Imade Y. Nsa, Kehinde T. Kareem

**Affiliations:** ^1^Department of Microbiology, University of LagosLagos, Nigeria; ^2^Institute of Agricultural Research and Training, Moor Plantation, Obafemi Awolowo UniversityIbadan, Nigeria

**Keywords:** cowpea, severity, virus, yield, interaction

## Abstract

This study was carried out to determine the effects of single infections and co-infections of three unrelated viruses on three cowpea cultivars (one commercial cowpea cultivar “White” and 2 IITA lines; IT81D-985 and TVu 76). The plants were inoculated with Cowpea aphid-borne mosaic virus (CABMV), genus *Potyvirus*, Cowpea mottle virus (CMeV), genus *Carmovirus* and Southern bean mosaic virus (SBMV), genus *Sobemovirus* singly and in mixture (double and triple) at 10, 20, and 30 days after planting (DAP). The treated plants were assessed for susceptibility to the viruses, growth, and yield. In all cases of infection, early inoculation resulted in higher disease severity compared with late infection. The virus treated cowpea plants were relatively shorter than buffer inoculated control plants except the IT81D-985 plants that were taller and produced more foliage. Single infections by CABMV, CMeV, and SBMV led to a complete loss of seeds in the three cowpea cultivars at 10 DAP; only cultivar White produced some seeds at 30 DAP. Double and triple virus infections led to a total loss of seeds in all three cowpea cultivars. None of the virus infected IITA lines produced any seeds except IT81D-985 plants co-infected with CABMV and SBMV at 30 DAP with a reduction of 80%. Overall, the commercial cultivar “White” was the least susceptible to the virus treatments and produced the most yield (flowers, pods, and seeds). CABMV was the most aggressive of these viruses and early single inoculations with this virus resulted in the premature death of some of the seedlings. The presence of the *Potyvirus*, CABMV in the double virus infections did not appear to increase disease severity or yield loss. There was no strong evidence for synergistic interactions between the viruses in the double virus mixtures.

## Introduction

Cowpea (*Vigna unguiculata* (L). Walp), accounts for a huge portion of the dietary protein of the people in sub-Saharan Africa (Li et al., 2001). Nigeria ranks first in cowpea production worldwide, and is responsible for about 61% of total world production of cowpea (IITA, 2009). Historically, cowpeas are known to produce a more satisfactory yield than most leguminous plants under a variety of climatic, soil, and cultural conditions. However, they are very susceptible to insect pests (Lephale et al., 2012) and many virus diseases (Karungi et al., 2000) that reduce yields. More than 140 viruses have been identified as naturally infecting cowpea (Hughes and Shoyinka, 2003) but only nine have been reported in Nigeria (Taiwo, 2003), and these are Cowpea aphid-borne mosaic virus (CABMV), Cowpea golden mosaic virus (CPGMV), Southern bean mosaic virus (SBMV), Sunhemp mosaic virus (SHMV), Blackeye mosaic virus (BICMV), Cucumber mosaic virus (CMV), Cowpea mottle virus (CMeV), Cowpea yellow mosaic virus (CPMV), and Cowpea mild mottle virus (CPMMV).

CABMV was first reported and described as a distinct *Potyvirus* infecting cowpea in Italy (Vidano and Conti, 1965; Lovisolo and Conti, 1966; Bashir et al., 2002), then in Nigeria in 1976 (Ladipo, 1976). It is of widespread in distribution in Nigeria and in other major cowpea growing regions of the world. It has also been reported from several African countries including, Kenya (Bock, 1973), Tanzania (Patel and Kuwite, 1982), Botswana (Burke et al., 1986), Uganda (Orawu et al., 2005). CABMV is the most important viral pathogen of cowpea in Nigeria, as far as pathogenic variation and yield losses are concerned (Raheja and Leleji, 1974; Thottappilly and Rossel, 1992; Shoyinka et al., 1997). It is readily transmissible by sap inoculation and by several aphids in a non-persistent manner (Vidano and Conti, 1965; Atiri, 1982; Atiri et al., 1984). It is also seed-borne in cowpea, but transmission is dependent upon cowpea cultivar and virus strain (Aboul-Ata et al., 1982; Gumedzoe, 1985). It has a wide experimental host range including many species in the Leguminosae, Chenopodiaceae, Curcubitaceae, Solanaceae, and Amaranthaceae (Lovisolo and Conti, 1966; Bos, 1970; Bock, 1973). Virus may induce local lesions or systemic infections depending on the host, infected plants show dark green vein–banding, leaf deformation, crinkling, blistering, and stunting (Bock and Conti, 1974). However, the severity of the symptoms depends on the interaction between the host cultivar and virus strain (Rossel and Thottappilly, 1985).

Field occurrence of SBMV on cowpea has also been reported from the world, U.S.A. (Kuhn, 1963), India (Singh and Singh, 1974), Ghana (Lamptey and Hamilton, 1974), Ivory Coast (Fauquet and Thouvenel, 1980), Senegal (Gaikward and Thottappilly, 1988), Togo (Gumedzoe et al., 1989), and Nigeria (Shoyinka et al., 1979). The systemic symptoms induced by SBMV include vein clearing, followed by a mild to severe mottling or coarse mosaic pattern (Shepherd and Fulton, 1962; Allen et al., 1981). It is transmissible by inoculation of sap, beetle, and is seed borne (Tremaine and Hamilton, 1983).

CMeV was first described from Nigeria, where it was isolated from Bambarra groundnut (*Vigna subterranea*) and cowpea, *V. unguiculata* (Robertson, 1963, 1966). In Nigeria, CMeV is commonly found in the southern rainforest and guinea savannah zones where most of the Bambara groundnut is grown (Rossel and Thottappilly, 1985). It has also been reported from other West African countries, Ivory Coast (Thouvenel, 1988), Togo (Gumedzoe et al., 1989), and the Republic of Benin (Thottappilly and Rossel, 1988). In tolerant cowpea varieties, the symptom of this virus consists basically of mottling; whereas in severe infections, CMeV may induce leaf distortion, reduction in leaf size and witches broom syndrome. CMeV is transmitted principally by a chrysomelid beetle vector, *Ootheca mutabilis* (Shoyinka et al., 1978) and by sap inoculation.

In Nigeria, there have been reports of single and multiple (double and triple) virus infections of cultivated cowpeas resulting in complete yield loss (Raheja and Leleji, 1974; Kareem and Taiwo, 2007; Taiwo et al., 2007). Mixed virus infection of plants often results in intensified symptom severity than those caused by each individual virus, and higher virus accumulation, a phenomenon referred to as synergy (Wang et al., 2002; Untiveros et al., 2007). Synergism occurs in mixed infections when a pair of the viruses is unrelated, with the overall effect of the viruses eliciting more severe disease symptoms in the host plant than those produced by each of the viruses separately (Cho et al., 2000; Murphy and Bowen, 2006). Examples of synergistic viral diseases have been recorded for many decades (Shi et al., 1996). The severity of the disease symptoms is also greatly increased if one of the infecting viruses is a member of the genus *Potyvirus* (Pio-Ribeiro et al., 1978). Nevertheless, not all combinations of unrelated viruses result in increased symptoms (Anjos et al., 1992). Shoyinka et al. (1978) reported that CMeV frequently occurred in mixed infections with Cowpea yellow mosaic virus (CYMV) or SBMV in cowpea fields located in southwestern Nigeria.

One method of detecting viruses is by biological properties (Naidu and Hughes, 2003). In this study, two International Institute of Tropical Agriculture (IITA) cowpea lines (IT81D-985, TVu 76) and a local commercial cultivar (White) were evaluated for symptom severity, growth defects, and yield losses as a result of single and mixed infections of CABMV, CMeV, and SBMV. We also ascertained whether the qualitative observation of increased symptom severity, reductions in growth and yield presumably attributed to synergistic interactions of the viruses could be further validated by quantitative methods. Serological tests were done to determine relative virus concentration.

## Materials and methods

### Source and cultivation of cowpea lines/cultivars

The cowpea lines, TVu 76 and IT 81D-985 used in this study were obtained from the Germplasm Resource Unit of the International Institute of Tropical Agriculture (IITA), Ibadan and the commercial “White” cultivar was purchased from Sandgrouse Market, Lagos. The IT81D-985 is a medium-late maturing semi spreading cowpea line (Singh, 2011) and line TVu 76 is known to be susceptible to leafhoppers (Raman et al., 1980). The cowpea lines/cultivars were cultivated as described by Kareem and Taiwo (2007).

### Source and maintenance of viruses

The viruses used in this research were Cowpea aphid-borne mosaic virus genus, *Potyvirus*; Southern bean mosaic virus, genus *Sobemovirus*; and Cowpea mottle virus, genus *Carmovirus*. The viruses were obtained from infected cowpea plants or tissue stored over calcium chloride at the IITA Ibadan. These viruses were maintained on the Ife Brown variety in the Greenhouse of the Botanical Garden of University of Lagos (Kareem and Taiwo, 2007).

### Inoculation of cowpea cultivars with viruses

Seedlings were mechanically inoculated at 10, 20, and 30 days after planting. Each pot was labeled according to the type of virus treatment, including negative controls, name of cultivar and date of inoculation. Test plants were dusted with carborundum (180 mesh) to increase entry of the virus into the hosts. Single virus inoculum was prepared by grinding virus-infected leaves with a sterile mortar and pestle at the rate of 1 g of tissue to 2 ml of buffer. However, mixed viral treatments were obtained by mixing saps from the relevant inocula at ratio 1:1 (V/V) just before inoculation. The pestle was used to rub sap over the upper leaf surface from base to the top of the leaf. After inoculation, the leaves were rinsed immediately with water to prevent the harmful effects of the sap. Inoculated plants were kept in the University of Lagos Greenhouse. The different treatments are listed below.

- Single virus inoculation
  Each of the cultivars: TVu 76, IT81D-985 and commercial cultivar “White” was inoculated singly with CABMV, CMeV, or SBMV.
- Double virus inoculation
  Each of the three cowpea lines/cultivar was inoculated with double virus mixtures i.e., CABMV + CMeV, CABMV + SBMV, and CMeV + SBMV.
- Triple virus inoculation
  Each of the three cowpea lines was also inoculated with a combination of the three viruses, i.e., CABMV + SBMV + CMeV.
- Mock inoculation with buffer
  Control plants of each of the cowpea lines/cultivars were inoculated with buffer only. The buffer was prepared by dissolving 17.4 g of di-potassium hydrogen orthophosphate (K_2_HPO_4_) in one liter (1l) distilled water (0.1 M solution); 0.1 M potassium di-hydrogen orthophosphate (KH_2_PO_4_) was added (3.4 g of KH_2_PO_4_ dissolved in 250 ml) to achieve a pH 7.5 (Walkey, 1985). The working concentration was 0.05 M.

### Inoculation procedure

- First stage inoculation
  The first inoculation was done 10 days after planting (DAP). The sap from the different virus treatments above or buffer was rubbed onto the plant surface of the fully expanded leaves of each of the three cultivars. Symptoms were observed at 10, 20, and 30 days after inoculation (DAI).
- Second stage inoculation
  The second inoculation was done 20 DAP. The sap from the different virus treatments or buffer was rubbed on the leaves of each of the three cultivars. Symptoms were observed at 10, 20, and 30 DAI.
- Third stage inoculation
  The second inoculation was carried out 30 days after planting. Symptoms were observed at 10, 20, and 30 DAI. After each inoculation, plants were rinsed with water to reduce the effect of caborundum on leaves that may interfere with photosynthesis. Plants were kept in the greenhouse under ambient temperatures ranging from 25 to 28°C, watered every other day, and observed for symptom development.

### Experimental design

The experimental design used in the greenhouse was randomized complete block design (RCBD). There were three cowpea lines/cultivars, three replicates, three different stages of virus inoculation and eight virus treatments including the negative control (3 × 3 × 3 × 8) making a total of 216 pots.

### Effect of virus treatments on disease severity

The reaction of the cultivars to mechanical inoculation with the viruses (single and multiple) and buffer was determined by rating inoculated cowpea plants based on symptom development at 10, 20, and 30 DAI. The rating was done on a scale of 1 to 5, where 1, no symptoms; (resistant) 2, slight mosaic/mottle on leaves (susceptible); 3, moderate mosaic/mottle/blistering/curling of leaves (susceptible); 4, severe mosaic/mottle/blistering, leaf reduction (susceptible); 5, very severe symptoms leading to stunting, apical necrosis, and plant death.

### Effects of virus treatments on growth traits

The effect of virus treatments and buffer on the plant height, number of leaves of White, IT81D-985 and TVu 76, was determined at 10 DAP 10 DAI and 30 DAP 10 DAI. The plant height was measured with a ruler in cm. and the leaves were counted. The percentage reduction in plant height, and number of leaves in virus-infected plants was calculated as:

$$\%\text{ reduction in plant height}=[({\text{H}}_{\text{B}}-{\text{H}}_{\text{V}})/{\text{H}}_{\text{B}}]\times 100$$

Where:

H_B_ = plant height of buffer inoculated plant

H_V_ = plant height of virus inoculated plant

$$\%\text{ reduction in leaf number}=[({\text{L}}_{\text{B}}-{\text{L}}_{\text{V}})/{\text{L}}_{\text{B}}]\times 100$$

Where:

L_B_ = number of leaves of buffer inoculated plant

L_V_ = number of leaves of virus inoculated plant

### Effect of virus treatments on yield parameters of the cowpea varieties

The effect of virus treatments and buffer on the number of flowers, number of pods, pod length, seed number, and seed weight was determined. Pod length was measured with a ruler and the seeds were weighed on a Mettler -Toledo balance (Columbus, OH, U.S.A).

### Determination of the type of biological response among the viruses

To determine the type of biological interactions existing between the viruses in double co-infections, we compared the growth traits of cowpea plants exposed to double virus treatments to the single virus inoculated plants. We calculated the degree of interactions of the viruses in the mixed virus treatments using the Abbott's formula (Abbott, 1925; Gisi, 1996).

Cexp = A + B−(AB/100)

Where Cexp is the expected level of disease, and A and B are corresponding responses due to infection by virus A and B, respectively, as observed in the experiment, AB is the response of the double virus infection (Murphy and Bowen, 2006). This was done for all the double infections, CABMV + CMeV, CABMV + SBMV, and CMeV + SBMV of the three cowpea varieties under investigation.

The means of the different growth traits were used to quantitatively determine the degree of the observed reduction. Plant height and leaf number were the two traits considered. Rather than using the strict mathematical definition of a synergistic interaction as the ratio of the observed response (Cobs) to the expected response (Cexp) if greater than 1.0, additive, if equal to 1 and antagonistic, if less than 1. We adopted the cut offs by Gisi et al. (1985), where a biological response can be categorized as follows: (1) synergistic, if the interaction of the ratio of the observed response (Cobs) to the expected response (Cexp) is greater than 1.5, (2) additive, between 0.5 and 1.5, and (3) antagonistic, less than 0.5.

### Data analysis

All the data obtained were analyzed using Statistical Package for Social Scientists (SPSS) version 16.0 and Duncan Multiple Range Test was used to compare means obtained at 5% level of probability (Little and Hills, 1972).

### Effect of virus treatment on relative virus concentration

Leaf samples from cultivar White, IT81D-985 and TVu 76 that received the different treatments were taken at 10 and 30 days after inoculation at the different stages of growth to determine virus concentration. The relative concentration of virus in single and mixed infections was determined serologically with antigen coated plate enzyme linked immunosorbent assay (ACP ELISA). The ELISA protocol was carried out as described as Taiwo et al. (2007).

## Results

### Symptomatology

The three cultivars/lines were susceptible to the three viruses used in this investigation. The inoculated leaves were observed for symptom development at different stages of growth after inoculation and recorded. The symptoms observed at 20 DAP were not intermediary or significantly different from those observed at 30 DAP (data not shown).

### Symptom severity of virus treatments on cowpea (white)

**Figure 1:**
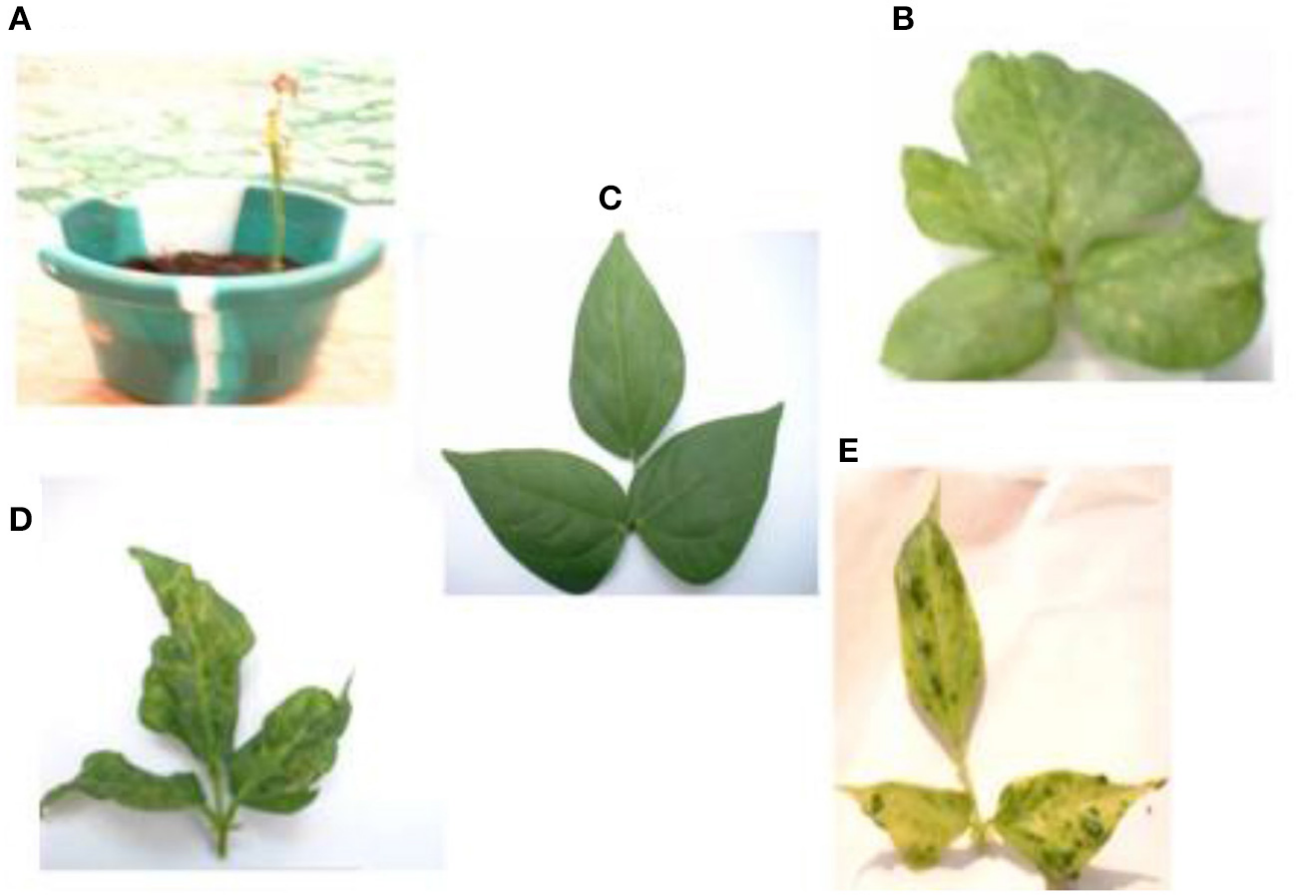
**Viral symptoms induced on the local commercial cultivar White**. **(A)** Apical necrosis and defoliation induced by triple virus treatment, CABMV + CMeV + SBMV that eventually led to plant death. **(B)** Leaf deformation and mosaic symptoms induced by double virus infection, CABMV + CMeV. **(C)** Buffer inoculated healthy fully expanded trifoliate leaves. **(D)** Mottling, leaf reduction and crinkling induced by SBMV. **(E)** Blistering and leaf chlorosis induced by CABMV.

The mixture of the triple viruses, CABMV + CMeV + SBMV induced the most symptoms. Its common symptoms were mosaic, mottling and especially apical necrosis that eventually led to plant death (Figure 1A). The triple virus treatments caused death at all stages of plant growth regardless of the time of inoculation. As for the double virus infections, only early inoculations (10 DAP) resulted in a severity score of 5 indicating that the plants died due to early infection (Table 1). Leaf deformation was observed in the mixture of CABMV and CMeV (Figure 1B). No symptoms were observed on the leaves of the buffer inoculated plants (Figure 1C).

**Table 1:** **Severity of single and multiple virus treatments on cowpea varieties**.

**Virus treatment**	**DAP *^x^***	**10 *^y^***			**20 *^y^***			**30 *^y^***		
		**White**	**IT81D-985**	**TVu 76**	**White**	**IT81D-985**	**TVu 76**	**White**	**IT81D-985**	**TVu 76**
CABMV	10	3^c^	3^c^	4^d^	2.5^bc^	5^e^	3^c^	5^e^	5^e^	4^d^
	30	2^b^	2^b^	1^a^	2^b^	3^c^	1^a^	2.5^bc^	4^d^	2^b^
CMeV	10	2^b^	2^b^	4.5^de^	3.5^cd^	2^b^	3.5^cd^	3.5^cd^	4^d^	4.5^de^
	30	1^a^	2^b^	2^b^	2.5^bc^	2^b^	2^b^	3.5^cd^	3.5^cd^	4.5^de^
SBMV	10	2.5^bc^	2^b^	2^b^	3^c^	2^b^	4^d^	3.5^cd^	5^e^	4^d^
	30	2^b^	2^b^	2^b^	2^b^	2^b^	2^b^	3^c^	2^b^	2^b^
CABMV + CMeV	10	2.5^bc^	4^d^	4.5^cd^	5^e^	2^b^	5^e^	5^e^	5^e^	5^e^
	30	2^b^	2^b^	2^b^	2^b^	2^b^	5^e^	5^e^	4^d^	5^e^
CABMV + SBMV	10	2.5^bc^	3.5^cd^	4^d^	5^e^	3.5^cd^	5^e^	5^e^	3.5^cd^	5^e^
	30	2^b^	3^c^	2^b^	2^b^	3^c^	4^d^	2.5^bc^	3.5^cd^	5^e^
CMeV + SBMV	10	2.5^bc^	2^b^	3.5^cd^	5^e^	3^c^	4.5^de^	5^e^	5^e^	5^e^
	30	2^b^	2^b^	2^b^	2^b^	2^b^	2^b^	2.5^bc^	3^c^	3^c^
CABMV + CMeV + SBMV	10	4.5^de^	4.5^de^	4^d^	4.5^de^	3.5^cd^	5^e^	5^e^	5^e^	5^e^
	30	2^b^	2^b^	2^b^	2^b^	2^b^	3^c^	5^e^	5^e^	3.5^cd^
BUFFER (negative control)	10	1^a^	1^a^	1^a^	1^a^	1^a^	1^a^	1^a^	1^a^	1^a^
	30	1^a^	1^a^	1^a^	1^a^	1^a^	1^a^	1^a^	1^a^	1^a^

*Means followed by the same letter in each column are not significantly different according to Duncan's multiple range Test at P ≤ 5%*.

x = age of plant at inoculation in days;

y *= disease severity was observed at 10, 20, and 30 days after inoculation at growth stages 10 and 30 DAP. CABMV, Cowpea aphid-borne mosaic virus; CMeV, Cowpea mottle virus; SBMV, Southern bean mosaic virus*.

For the single viruses, SBMV induced mostly mosaic and leaf curling symptoms but did not cause the death of any plants (Figure 1D). CABMV was the most aggressive of the viruses in this variety. Early inoculations with this virus resulted in premature death of some of the seedlings giving a severity score of 5 (Table 1). Its common symptoms included mosaic, vein -banding, chlorosis, blistering (Figure 1E) and stunting. Whereas, single infections by CMeV, induced mostly mottling and defoliation. The leaves of the buffer inoculated plants/controls were healthy, remained fully expanded and symptomless; the other plant parts also showed no disease symptoms.

### Symptom severity of virus treatments on line IT81D-985

The most severe symptom induced by the triple virus infection of CABMV + CMeV + SBMV on IT81D-985 was apical necrosis. The symptoms induced on its primary leaves were reddish necrotic lesions. Double virus treatments involving CABMV + CMeV and CMeV + SBMV led to the maximum severity score of 5 at 10 DAP, while early inoculation with CABMV + SBMV resulted in the highest severity score of 3.5 (Table 2).

**Table 2:** **The effects of single virus, double infections of CABMV + CMeV treatments and buffer on plant height and number of leaves in the White cultivar, and TVu 76**.

**Growth Parameter**		**Plant Height (cm)**		**Number of leaves**	
		**White**	**TVu 76**	**White**	**TVu 76**
**TREATMENT**	**DAP** ^*x*^				
CABMV	10	19.19^d^	24.69^c^	3.44^d^	14.11^b^
	30	92.99^ab^	46.84^ab^	19.22^ab^	16.67^ab^
CMeV	10	27.09^d^	16.3^d^	4.33^d^	5.25^c^
	30	52.78^bc^	36.12^b^	13.67^b^	12.22^bc^
CABMV + CMeV	10	24.13^d^	16.08^d^	2.22^d^	6.33^c^
	30	98.64^ab^	34.14^b^	13.44^b^	12.33^bc^
BUFFER	10	50.09^bc^	34.43^b^	26.44^a^	14.78^b^
	30	102.44^a^	54.49^a^	27.11^a^	18.33^a^
**Percent reduction in growth parameters of virus treated plants compared with mock inoculated plants**					
CABMV	10	61.69	28.29	86.99	4.53
	30	9.22	14.04	29.10	9.06
CMeV	10	45.92	52.66	83.62	64.48
	30	48.48	33.71	49.58	33.33
CABMV + CMeV (Cobs)	10	51.83	53.30	91.60	57.17
	30	3.71	37.35	50.42	32.73
Cexp	10	79.28	66.05	97.87	71.93
	30	53.23	43.02	64.25	39.37
Cobs: Cexp	10	0.65	0.81	0.94	0.79
	30	0.07	0.87	0.78	0.83

*DAP^x^, Growth parameters were measured 10 days after inoculation at 10 and 30 DAP. The age of the plant at 10 DAP was 20 days old, at 30 DAP, the age of the plant was 40 days old. CABMV, Cowpea aphid-borne mosaic virus; CMeV, Cowpea mottle virus. Each value is the mean of 3 replicates. In each column, means followed by the same letter are not significantly different (P = 0.05) according to Duncan's multiple range tests. Cexp = A + B − AB/100, A stands for CABMV alone and B for CMeV alone. If Cobs: Cexp < 0.5, interactions are antagonistic, Cobs: Cexp 0.5–1.5, interactions are additive, Cobs: Cexp >1.5, interactions are synergistic*.

**Figure 2:**
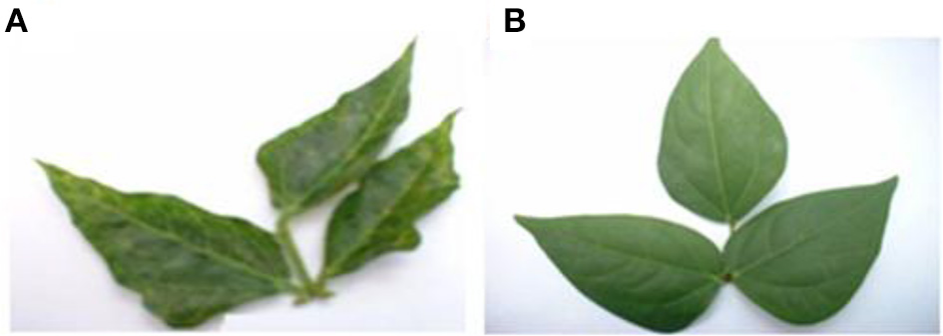
**(A)** Mottling and leaf reduction induced on line IT81D-985 by CMeV. **(B)** Symptomless buffer inoculated trifoliate leaf.

In this variety, single inoculations with CABMV or CMeV caused death in some of the plants at 10 and 30 DAP, respectively. CABMV induced vein- banding, mosaic, internode shortening, chlorotic lesions and apical necrosis. In addition to the characteristic mottling of CMeV (Figure 2), reduction in size of leaves, defoliation, and apical necrosis were also observed. The general symptoms induced by SBMV on this line included mosaic, leaf reduction, and defoliation. Inoculations with each of the double virus mixtures caused the death of some of the plants. While early inoculation with CMeV + SBMV at 10 DAP resulted in the death of some plants, as well as severe mottling among other symptoms. Single inoculation with either CABMV or SBMV produced a severity score of 5 that caused death in the plants at 10 DAP 10 DAI and 10 DAP 30 DAI. The severity score of 2 was observed in the early- inoculated CMeV plants, severity worsened to a score of 4 at 10 DAP 30 DAI. Mottling was observed on the leaves of cowpea inoculated with CMeV (Figure 2A) but not death. The symptoms induced on its primary leaves were reddish necrotic lesions but like in the “White” variety, CABMV also induced chlorotic lesions.

### Symptom severity of virus treatments on cowpea (TVu 76)

As observed in “White” and IT81D-985 varieties, triple infections resulted in chronic symptoms, most especially apical necrosis which eventually led to the death of some of the TVu 76 plants. Generally, all the virus treatments (whether single, double, or triple) induced defoliation, while the buffer inoculated plants remained healthy and symptomless.

Severity scores in plants with single virus treatments were significantly reduced at 30 DAP compared with 10 DAP. TVu 76 plants inoculated with CABMV only at 30 DAP, were tolerant at the initial stage (10 DAI) of infection but later showed mild symptoms with a score of 2 at 30 DAI. The most common symptom caused by CABMV was internode shortening. Single infections by CMeV induced the most symptoms including mottling and apical necrosis, while SBMV induced basal necrosis and leaf curling among other symptoms. The severity scores of CABMV + CMeV and CABMV + SBMV at 10 DAP were high (between 4 and 5) causing death of some plants (Table 1). At 30 DAP, CMeV + SBMV induced less symptoms than CABMV + CMeV and CABMV + SBMV treatments and did not cause plant death. The buffer inoculated plants had a score of 1 implying that the plants remained healthy and symptomless.

### CABMV was the most aggressive of single virus infections in the white cultivar but not in line TVu 76

To determine the effects of single virus treatments to the different cowpea, we exposed each cowpea variety to single treatments of the 3 viruses—CABMV, CMeV, and SBMV. Generally, the entire virus treatments at 10 DAP caused a significant reduction in plant height and leaf number in comparison with the controls (Tables 2–4). We observed that in the White cultivar, CABMV was the most aggressive but caused the least reduction in vegetative growth in TVu 76 (Table 2). In the single infection of the cowpea cultivar “White,” CABMV infected plants were the shortest with an average height of 19.19 cm, while the average height of the healthy was 50.09 cm. compared to the healthy's average of 26 leaves. In TVu 76, single infections with CMeV and SBMV caused similar percentage reductions in plant height and leaf number at 10 and 30 DAP (Table 4).

**Table 3:** **The effects of single virus, double infections of CABMV + SBMV treatments and buffer on plant height and number of leaves in the White cultivar and TVu 76**.

**Growth Parameter**		**Plant Height (cm)**		**Number of leaves**	
		**White**	**TVu 76**	**White**	**TVu 76**
**TREATMENT**	**DAP** ^*x*^				
CABMV	10	19.19^d^	24.69^c^	3.44^d^	14.11^b^
	30	92.99^ab^	46.84^b^	19.22^ab^	16.67^ab^
SBMV	10	21.43^d^	16.07^d^	8.11^c^	6.33^c^
	30	51.92^bc^	34.13^b^	23.67^ab^	12.33^bc^
CABMV + SBMV	10	26.24^d^	22.15^c^	4.89^d^	2.67^d^
	30	43.11^c^	34.44^b^	15.67^b^	4.22^cd^
BUFFER	10	50.09^bc^	34.43^b^	26.44^a^	14.78^b^
	30	102.44^a^	54.49^a^	27.11^a^	18.33^a^
**Percent reduction in growth parameters of virus treated plants compared with mock inoculated plants**					
CABMV	10	61.69	28.29	86.99	4.53
	30	9.22	14.04	29.10	9.06
SBMV	10	57.22	53.33	69.33	57.17
	30	49.32	37.36	12.69	32.73
CABMV + SBMV (Cobs)	10	47.61	35.67	81.51	81.94
	30	57.92	36.80	42.20	76.98
Cexp	10	83.61	66.53	96.01	59.11
	30	53.99	46.16	38.10	38.83
Cobs: Cexp	10	0.57	0.54	0.85	1.39
	30	1.07	0.80	1.11	1.98

*DAP^x^,- Growth parameters were measured 10 days after inoculation at 10 and 30 DAP. The age of the plant at 10 DAP was 20 days old, at 30 DAP, the age of the plant was 40 days old. CABMV, Cowpea aphid borne mosaic virus; SBMV, Southern bean mosaic virus. Each value is the mean of 3 replicates. In each column, means followed by the same letters are not significantly different (P = 0.05) according to Duncan's multiple range tests. Cexp = A + B − AB/100, A stands for CABMV alone and B for SBMV alone. If Cobs: Cexp < 0.5, interactions are antagonistic, Cobs: Cexp 0.5–1.5, interactions are additive, Cobs: Cexp > 1.5, interactions are synergistic*.

**Table 4:** **The effects of single virus, double infections of CMeV + SBMV treatments and buffer on plant height and number of leaves in the White cultivar and line TVu 76**.

**Growth Parameter**		**Plant Height (cm)**		**Number of leaves**	
		**White**	**TVu 76**	**White**	**TVu 76**
**TREATMENT**	**DAP** ^*x*^				
CMeV	10	27.09^d^	16.3^d^	4.33^d^	5.25^c^
	30	52.78^bc^	36.12^b^	13.67^b^	12.22^bc^
SBMV	10	21.43^d^	16.07^d^	8.11^c^	6.33^c^
	30	51.92^bc^	34.13^b^	23.67^ab^	12.33^bc^
CMeV + SBMV	10	19.48^d^	18.2^d^	4.33^d^	4.5^cd^
	30	87.48^b^	34.00^b^	20.56^ab^	16.56^ab^
BUFFER	10	50.09^bc^	34.43^b^	26.44^a^	14.78^b^
	30	102.44^a^	54.49^a^	27.11^a^	18.33^a^
**Percent reduction in growth parameters of virus treated plants compared with mock inoculated plants**					
CMeV	10	45.92	52.66	83.62	64.48
	30	48.48	33.71	49.58	33.33
SBMV	10	57.22	53.33	69.33	57.17
	30	49.32	37.36	12.69	32.73
CMeV + SBMV (Cobs)	10	61.11	47.14	83.62	69.55
	30	14.60	37.60	24.16	9.66
Cexp	10	76.86	77.90	94.98	84.79
	30	73.89	58.48	55.97	55.16
Cobs: Cexp	10	0.80	0.61	0.88	0.82
	30	0.20	0.64	0.43	0.18

*DAP ^x^, Growth parameters were measured 10 days after inoculation at 10 and 30 DAP. The age of the plant at 10 DAP was 20 days old, at 30 DAP, the age of the plant was 40 days old. CMeV, Cowpea mottle virus; SBMV, Southern bean mosaic virus. Each value is the mean of 3 replicates. In each column, means followed by the same letter are not significantly different (P = 0.05) according to Duncan's multiple range tests. If Cobs: Cexp < 0.5, interactions are antagonistic, Cobs: Cexp 0.5–1.5, interactions are additive, obs: Cexp >1.5, interactions are synergistic*.

### The presence of a potyvirus (CABMV) had no significant effect on severity of growth reduction in the double infections

On exposure to the different double virus combinations, CABMV + CMeV, CABMV + SBMV, CMeV + SBMV, there were no significant differences in the response of the White and TVu 76 varieties to the 3 types of double infections (Tables 2–4).

In the White variety at 10 DAP, although the mixture of CMeV and SBMV caused the most stunting with an average height of 19.48 cm, there was no significant difference in the average height and leaf number of the plants that received CABMV + CMeV or CABMV + SBMV or CMeV + SBMV. However, there were significant differences in these parameters when the plants received the virus treatment at 30 DAP.

All the TVu 76 plants that received virus treatments at 10 and 30 DAP were significantly shorter than the buffer inoculated plants. There was no significant differences in the effects of the 3 types of double virus infections on plant height at 10 and 30 DAP.

### Virus treatments promoted increase in foliage and plant height in the IITA line IT81D-985

Unexpectedly, some of the virus treated plants were taller and produced more leaves than the control plants. For the plants inoculated at 10 DAP, those that received the following treatments (i) CMeV (ii) CABMV + SBMV (iii) CABMV + CMeV were taller than the control plants and also produced more leaves. Considering those treated at 30 DAP, all the plants that received the virus treatments produced more leaves and were taller than the controls except those that were treated with SBMV alone (Table 5).

**Table 5:** **The effect of single virus, double virus infections and buffer on plant height and leaf number in IT 81D-985**.

**Treatment**	**DAP ^*x*^**	**Plant height (cm)**	**Leaf number**
CABMV	10	8.47^d^	4.33^d^
	30	50.80^ab^	13.89^b^
CMEV	10	26.33^c^	10.56^b^
	30	61.24^a^	19.33^a^
SBMV	10	6.17^d^	7.44^c^
	30	21.22^c^	17.89^a^
CABMV + CMeV	10	21.87^c^	8.78^c^
	30	36.98^b^	11.56^b^
CABMV + SBMV	10	23.42^c^	13.78^b^
	30	50.94^b^	15.56^a^
CMEV + SBMV	10	13.41^c^	3.78^d^
	30	75.77^ab^	18.33^a^
Buffer	10	19.33^c^	8.00^d^
	30	34.71^b^	8.56^c^

*DAP^x^, Growth parameters were measured 10 days after inoculation at 10 and 30 DAP. The age of the plant at 10 DAP was 20 days old, at 30 DAP, the age of the plant was 40 days old. CABMV, Cowpea aphid borne mosaic virus. CMeV, Cowpea mottle virus; SBMV, Southern bean mosaic virus. Each value is the mean of 3 replicates. In each column, means followed by the same letter are not significantly different (P = 0.05) according to Duncan's multiple range tests*.

### Co-infection with triple viruses did not have a greater impact than double virus infections on growth traits

To assess if the extent of damage caused by mixed infections could be greater if there were more than 2 viruses involved, we looked at the effects of the triple virus treatment CABMV + CMeV + SBMV on the three cowpea varieties (Table 6). In all the cultivars/lines, the triple virus treated plants were significantly shorter than the control plants and produced less leaves. In White, there was no difference between the effects of the double and triple virus infections. However, the double infections were more aggressive than the triple virus infections in TVu 76 (Tables 2–4, 6).

**Table 6:** **The effect of co-infection with a mixture of the three viruses/buffer and age of plant at inoculation on growth parameters on commercial cultivar White and IITA lines IT81D-985 and TVu 76**.

**Virus Treatment**	**DAP ^*x*^**	**PLANT HEIGHT**			**LEAF NUMBER**		
		**White**	**IT81D-985**	**TVu 76**	**White**	**IT81D-985**	**TVu 76**
CABMV + CMeV + SBMV	10	27.15^d^	13.82^c^	21.17^c^	3.22^d^	3.89^d^	10.111^bc^
	30	58^bc^	40.07^ab^	36.12^b^	12^b^	6.89^c^	3.11^d^
BUFFER	10	50.09^bc^	19.33^c^	34.43^b^	26.44^a^	8^c^	14.78^b^
	30	102.44^a^	34.71^b^	54.49^a^	27.11^a^	8.56^c^	18.33^a^

*DAP^x^, Growth parameters were measured 10 days after inoculation at 10 and 30 DAP. The age of the plant at 10 DAP was 20 days old, at 30 DAP, the age of the plant was 40 days old. CABMV, Cowpea aphid borne mosaic virus; CMeV, Cowpea mottle virus; SBMV, Southern bean mosaic virus. Each value is the mean of 3 replicates. In each column, means followed by the same letter are not significantly different (P = 0.05) according to Duncan's multiple range tests*.

### None of the early virus inoculated plants (10 DAP) produced any flowers, pods, or seeds

In all the three varieties, none of the virus treated plants produced any flowers, pods or seeds at 10 DAP. However, the plants inoculated with buffer only, produced flowers and seeds. For those inoculated at 30 DAP, all the virus treated cultivar White plants produced an average of 1–4 flowers compared to the 7 flowers produced by the healthy. CABMV + SBMV, as well as CABMV + CMeV + SBMV treated ones produced the least—an average of 1 flower. All of the infected plants produced pods and seeds except those that received the CABMV + CMeV + SBMV and CABMV + SBMV treatments.

The pods from the CABMV + CMEV treated plants were the shortest at 2.13 cm whereas the average length of the pods from the negative controls was 9.27 cm. CMeV treated plants had the least yield of approximately 1 seed, while the CABMV treated plants as well as SBMV treated plants had the same average yield of 3 seeds. CMeV + SBMV treated plants had the most yields for the double infections with an average seed number of 2. Reduction in seed number ranged from 47 to 89%. The average unit weight of the seeds produced from the virus treated plants was less than that produced by the control plants (0.15 g) except in those inoculated with CMeV + SBMV. CABMV treated plants produced the smallest seeds (0.09 g), while the average weight of seeds from CMeV + SBMV treated ones was 0.21 g.

For the IT81D-985 inoculated at 30 DAP, only those treated with CABMV singly, CABMV + CMeV and CABMV + SBMV and the healthy produced flowers. Of the virus treated plants that produced flowers, only those treated with CABMV + SBMV produced pods, which were one-third the length of those produced by the healthy. The average number of seeds they produced was 2 compared to about 5 produced by the control plants. The average unit weight of seeds from the healthy plants was at least 4 times the weight of the seeds from infected plant (Table 7).

**Table 7:** **The effect of virus/buffer treatments and age of plant at inoculation on yield parameters on commercial cultivar White and IITA lines IT81D-985 and TVu 76**.

**Virus Treatment**	**^*X*^**	**Flower number**			**Pod number**			**Pod length**			**Seed number/pod**			**Seed weight (g)**		
		**White**	**IT81D-985**	**TVu 76**	**White**	**IT81D-985**	**TVu 76**	**White**	**IT81D-985**	**TVu 76**	**White**	**IT81D-985**	**TVu 76**	**White**	**IT81D-985**	**TVu 76**
CABMV	10	0	0	0	0	0	0	0	0	0	0	0	0	0	0	0
	30	2.67^c^	0.33^b^	2.33^b^	0.67^c^	0	0	5.2^b^	0	0	3^ab^	0	0	0.09^c^	0	0
CMeV	10	0	0	0	0	0	0	0	0	0	0	0	0	0	0	0
	30	2.33^c^	0	1.67^b^	1.33^bc^	0	0	6.33^b^	0	0	0.67^c^	0	0	0.11^b^	0	0
SBMV	10	0	0	0	0	0	0	0	0	0	0	0	0	0	0	0
	30	3.3^b^	0	0	2.67^b^	0	0	7.03^b^	0	0	3^ab^	0	0	0.09^c^	0	0
CABMV + CMeV	10	0	0	0	0	0	0	0	0	0	0	0	0	0	0	0
	30	3.67^b^	0.33^b^	0	1.67^bc^	0	0	2.13^c^	0	0	1.33^b^	0	0	0.05^c^	0	0
CABMV + SBMV	10	0	0	0	0	0	0	0	0	0	0	0	0	0	0	0
	30	1^cd^	3^ab^	0	0	0.67^b^	0	0	3.57^b^	0	0	2^b^	0	0	0.04^b^	0
CMeV + SBMV	10	0	0	0	0	0	0	0	0	0	0	0	0	0	0	0
	30	4^b^	0	0	3.3^b^	0	0	5.57^b^	0	0	1.87^b^	0	0	0.21^a^	0	0
CABMV + CMeV + SBMV	10	0	0	0	0	0	0	0	0	0	0	0	0	0		0
	30	1^cd^	0	0	0	0	0	0	0	0	0	0	0	0	0	0
BUFFER	10	6.33^a^	4^a^	7.33^a^	5.67^ab^	3.3^a^	1.00^b^	10.03^a^	6.27^b^	8.43^b^	4.67^a^	4.67	5	0.15^b^	0.1768	0.1403^b^
	30	7^a^	3.67^a^	2^b^	6.3^a^	2.67^a^	1.33^a^	9.27^a^	10.1^a^	9.23^a^	4	4.67	5	0.15^b^	0.178^a^	0.1423^a^

*DAP^x^, Growth parameters were measured 10 days after inoculation at 10 and 30 DAP. The age of the plant at 10 DAP was 20 days old, at 30 DAP, the age of the plant was 40 days old. CABMV, Cowpea aphid borne mosaic virus. CMeV, Cowpea mottle virus; SBMV, Southern bean mosaic virus. Each value is the mean of 3 replicates. In each column, means followed. Each value is the mean of 3 replicates. In each column, means followed same letter are not significantly different (P = 0.05), and all zero values carry the same letter according to Duncan's multiple range tests*.

Of the TVu 76 plants that received virus treatments at 30 DAP, only the plants infected singly with CABMV or CMeV produced flowers. All the others did not produce any flower or pod. None of the virus treated TVU plants produced any seeds. The control plants produced flowers, pods and seeds (Table 7).

### Co-infection with triple viruses resulted almost in a total loss in yield loss

The triple virus infections resulted in a total loss of flowers, pods, and seeds except for the one flower produced by cultivar White. With regards to yield loss, the triple virus infections caused the most damage.

### Relative concentration of the viruses in the cowpea lines

The ELISA test was also used to establish the virus concentration in the cowpea cultivar White, IITA lines- IT81D-985 and TVu 76 infected singly by CABMV, CMEV, and SBMV and in mixtures. The samples were obtained from the inoculated leaf samples at 10 and 30 DAP. The ELISA results showed that the cowpea cultivar White and TVu 76 were susceptible to the each of the viruses treatments (Taiwo et al., 2007) and the mock inoculated plants were virus free.

**Figure 3:**
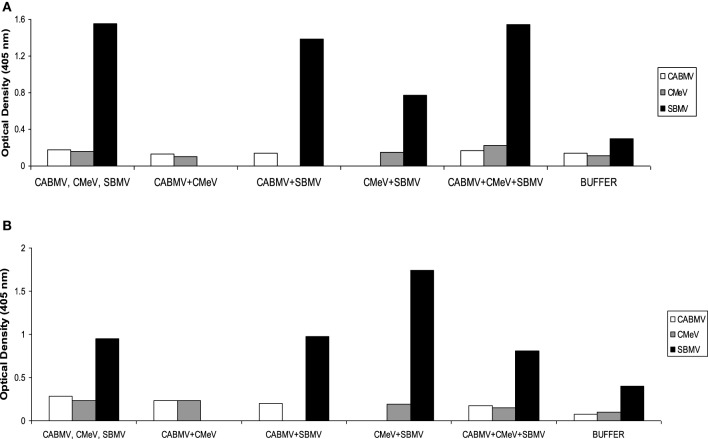
**Optical density readings (absorbance values) representing relative virus concentrations in leaf samples from IITA cowpea line IT81D-985 inoculated with single and mixed viruses at (A) 10 DAP (B) 30 DAP**. The virus titres were determined by ACP ELISA. Values are averages of samples taken at 10 and 30 days after inoculation.

For IITA line, IT 81D-985, only SBMV was detected at reasonable concentrations in the applicable treatments. In the single virus treatments, the absorbance values of CABMV and CMeV were 0.1805 and 0.155; these values were not significantly different from their negative controls of 0.1355 and 0.113, respectively. In the double infections and triple infections, the virus titres of CABMV and CMeV were also not significant to be considered positive. For the sample to be considered positive for virus presence, it must have at least twice the absorbance value of negative control. The optical density readings are shown in Figure 3.

### Lack of strong evidence for synergistic interactions among the unrelated viruses in the mixed infection

Based on our preliminary statistical analysis, the effects on the growth parameters did not suggest synergistic interactions. To validate our observation on the absence of synergism, we quantified using the Abbott's formula and set the boundaries for the type of response according to Gisi et al. (1985) instead of using the strict mathematical cut offs of synergism as being defined as Cobs: Cexp > 1 (Gisi, 1996). From the calculations, the reductions in the vegetative traits are not indicative of synergism in White and TVu 76 except the reduction in leaf number by CABMV + SBMV in TVu 76 (Table 3). The dual infections in IT81D-985 resulted in an increase in plant height and number of leaves; therefore, we could not make any calculations for this line (Table 5). The data is more indicative of an additive relationship between these viruses in White and TVu 76 (Tables 2–4).

## Discussion

Natural infections of cowpea grown in Nigeria by Cowpea aphid-borne mosaic virus (CABMV), Southern bean mosaic virus (SBMV), and Cowpea mottle virus (CMeV) and others have been reported by Shoyinka et al. (1997). Comparative studies of single and mixed unrelated virus infections of cowpea have been done (Pio-Ribeiro et al., 1978; Owolabi et al., 1988; Shoyinka et al., 1997; Martin et al., 2004; Orawu et al., 2005; Kareem and Taiwo, 2007; Taiwo et al., 2007; Akinjogunla et al., 2008).

This study showed that the single and multiple virus infections have significant effects on the three Cowpea varieties investigated. The three Cowpea varieties- Commercial cultivar “White,” IITA Lines IT 81D-985 and TVu 76 were susceptible to the single and mixed infections of Cowpea aphid-borne mosaic virus (CABMV), Southern bean mosaic virus (SBMV), and Cowpea mottle virus (CMeV). The common symptoms induced by the virus infections were mosaic, mottling, vein banding, chlorosis, and stunting. The investigation confirmed that triple infections in the three varieties produced more severe visual symptoms than the double or single infections. Severity scores ranging from 2 to 5 were recorded for the cowpea cultivars infected with the viruses. The high scores might be due to the fact that cowpea is the primary host of these viruses. The severity of the virus diseases varied depending on the cowpea variety, the type of treatment and age at the onset of virus treatment. The severity of virus infection was higher at 10 DAP than at 30 DAP; the severity also increased from 10 DAI to 30 DAI. In line with this, Uyemoto et al. (1981) reported that early infection of plants by viruses results in a more drastic response than infections at an advanced stage. Kareem and Akinjogunla (2008) had also previously reported that the infection of cowpea with three unrelated viruses resulted in increased symptom severity at early stage of plant growth. The reason for this is that at an early stage of growth, cultivars might not have built up enough defense mechanism to combat diseases.

Generally, all the virus treatments caused some degree of reduction in vegetative growth and total yield. The virus-infected plants were shorter than buffer inoculated plants due to shorter internodes with fewer leaves. However, in IT81D-985, an anomaly was observed as some of the virus treated plants produced more leaves than the control and were taller. In spite of this, there was hardly any yield from this cultivar. This means that the increase in the number of leaves and plant height was due to the reaction to the virus presence. On the other hand, the most common symptom in TVu 76 was defoliation. There was also hardly any yield from the infected TVu 76 plants. The commercial cultivar, White was the least susceptible in terms of yield. The different responses could be due to the varying tolerance level of the cultivars. Our results show that virus diseases can damage an entire crop, leaves, stems, flowers, and seeds in these varieties under investigation.

The effect of CABMV and CMeV on “White” and TVu 76 led to at least 60% reduction in leaf number and ultimately caused the withering of all the leaves inoculated at 10 days after planting. Our results also did not show that the double virus infections had a more severe effect than the single virus infections. This supports the report of Anjos et al. (1992) that not all the combinations of unrelated viruses result in increased symptoms. In some cases, the single virus had a more devastating effect on the crop than double infections involving that same virus. For example, the IT81D-985 plants doubly infected with CABMV and SBMV were the only ones that produced seeds. Wells and Deba (1961) reported a similar occurrence of 100% loss in yield in cowpea due to the single infection of Cowpea mosaic virus (CpMV). Raheja and Leleji (1974) had also reported that CABMV caused a complete loss of yield of commercial cowpea.

Infections at 10 DAP with CABMV in “White,” IT81D-985 and TVu 76 resulted in the death of some of the plants but not in those infected at 30 DAP. Of the 3 Cowpea varieties, the contrast in the impact of early and late infections on yield was most pronounced in the “White” variety. In the White variety, with the exception of plants that received the mixture of CABMV and SBMV, all the plants infected at 30 days after planting produced seeds unlike those infected at 10 DAP (Table 7). The results also correlate with previous information that the most severe effect on yield occurs as a result of early infection (Gay and Winstead, 1970; Kareem and Taiwo, 2007). This trend was also observed in the growth traits, as the heights of some “White” plants infected at 30 DAP were not significantly different from the height of the control plants. This compares favorably with the results of investigation by Owolabi et al. (1988), who reported that Cultivar “Ife Brown” plants inoculated with CpMV alone and in combination with BlCMV at the initiation of flowering showed no significant difference in yield compared with the control. A similar trend was also observed in TVu 76. Despite the observation that the vegetative growth in IT81D-985 was an aberration from the other two, some of the IT81D-985 plants showed this trend.

It was also observed that the response of the cowpea varieties to virus infections differed from one variety to another. While the major symptom of virus treated TVu 76 was defoliation, the response of the IT81D-985 plants to infection was increased leaf production. IT81D-985 is therefore sturdier than TVu 76 in response to virus infections. It is also significant that IT81D-985 was able to overcome infection by CABMV + SBMV and produced seeds, whereas, the White variety had no yield with the same treatment. The White variety shows similar characteristics to another local variety, Oloyin, reported by Kareem and Taiwo (2007) in that it was the most resistant of the three cultivars/varieties and produced seeds at 30 DAP.

Our results for growth and yield parameters for uninfected (buffer inoculated) IT81D-985 plants compare favorably with a previous report by Ekpo et al. (2012). They had evaluated plant height and leaf number of IT 81D-985 among other cowpea lines. In their report, at 21 days, plant height of IT81D-985 was 18.4 ± 1.38 cm and at 14 days, the number of leaves was 8.42 ± 0.2, our average value at 20 days old for negative controls were 19.33 cm and 8 for plant height and number of leaves, respectively. However, at advanced stages of plant growth, our findings were different from Ekpo et al. (2012). At 42 days old, they recorded an average plant height of 89.8 ± 13.72 cm and 18.25 ± 0.32 leaves, whereas at 40 days old, our healthy IT81D-985 plants measured an average of 34.19 cm and produced an average of 9 leaves. For yield parameters, the IT81D-985 plants inoculated with buffer at 10 DAP and 30 DAP produced pods with an average length of 6.27 and 10.1 cm, respectively, bearing similarity to the average pod length of 9.1 ± 2.26 cm recorded by Ekpo et al. (2012). Although our IT81D-985 plants inoculated with the buffer at 30 DAP did not grow as tall as expected, we ruled out the possibility that these group of plants did not grow optimally because serological evaluation showed that they were virus free and they had a good yield.

Serological detection of the three viruses in all the treatments for White and TVu 76 validated that the symptoms and reductions in growth traits were due to virus presence. However, in line IT81D-985 virus treated plants, ELISA results showed substantial accumulation of SBMV but not significant concentrations of CABMV or CMeV. Based on serological analysis, line IT81D-985 appeared resistant to CABMV and CMeV. In spite of the symptomatic IT81D-985 leaf samples having low titres of these two viruses, and more vegetative growth, there was still a significant reduction in yield.

Although Shoyinka et al. (1978) reported that the response of plants most times to mixed infections is more of synergism, and Pio-Ribeiro et al. (1978) reported that the symptoms induced by mixed infection in California Black eye by cucumber mosaic virus and BICMV were more severe and distinct from the relatively mild symptoms caused by either of the viruses, it is clear from our results that the interaction of the viruses in the double virus infections could not be suggestive of synergism, because the overall effect of these mixed treatments was not stronger than the sum of the effects caused by each of the viruses separately in all the cowpea varieties. The quantitative analysis of the growth parameters provided evidence for an additive relationship in the double virus infections in cultivar White and line TVu 76. With the unexpected results of increase in plant height and number of leaves of virus treated IT81D-985, in comparison with the healthy plants, we could not assess this line for synergistic interactions of viruses as in cultivar White and TVu 76.

It has been well documented for decades that diseases caused by viruses have been responsible for great damage, causing serious losses in cowpea crop yield in several countries (Lima et al., 1979; Damiri et al., 2013). It is also evident that our observations here, agree with earlier findings of Lana and Adegbola (1977), who reported that economic injury to cowpea due to virus infection depends on three factors- virus isolate, tolerance/resistance of the infected Cowpea cultivar and most importantly the age of the host plant at the time of infection. In summary, the results obtained confirm that plant virus infections cause the decline of cowpea plants, lower product quality, and ultimately result in crop losses.

The basic approaches for the control of viruses have not been overly successful because of non-curable nature of viral infections, lack of resistant cultivars with good agronomic quality and diversity of viruses and their natural vectors (Lecoq, 1998). The implications of these results are that there is a need to intensify efforts in developing advanced cowpea breeding lines/cultivars with multiple resistance to economically important viruses. With few virus control strategies available, pathogen-derived resistance is a promising tool in viral disease management. Since its first demonstration by Powell-Abel et al. (1986), it has been used with success against several other virus groups (Hull and Davies, 1992; Bau et al., 2003). It offers great potential to develop in a short time plants with highly durable resistance and broad efficacy (Kaniewski and Lawson, 1998). IITA reported that one of its 2003 milestones was the initiation of field testing of transgenic cowpea plants. Cowpea lines resistant to CABMV have been identified (Dhanasekar and Reddy, 2015).

The benefits of increased cowpea production include improved nutrition for humans and livestock, improved soil properties and substantial opportunities for greater income. The control of these viruses therefore is crucial to sustainable cowpea production most especially in sub-Saharan Africa.

### Conflict of interest statement

The authors declare that the research was conducted in the absence of any commercial or financial relationships that could be construed as a potential conflict of interest.
